# Applying the Quadrant Method for Pumping‐Trace Metal Correlations in Variable Time, Low‐Data Systems

**DOI:** 10.1111/gwat.13458

**Published:** 2024-12-20

**Authors:** Zachary D. Tomlinson, Kato T. Dee, Megan E. Elwood Madden, Andrew S. Elwood Madden

**Affiliations:** ^1^ School of Geosciences University of Oklahoma 100 East Boyd Street, RM 710 Norman OK 73019

## Abstract

Due to increasing global demand for fresh water, it is increasingly necessary to understand how aquifer pumping affects groundwater chemistry. However, comprehensive predictive relationships between pumping and groundwater quality have yet to be developed, as the available data, which are often collected over inconsistent time intervals, are poorly suited for long‐term historical correlation studies. For example, we needed an adequate statistical method to better understand relationships between pumping rate and water quality in the City of Norman (OK, USA). Here we used the interval‐scaled change in mean pumping rate combined with the Quadrant method to examine correlations between pumping rates and changes in trace metal concentrations. We found that correlations vary across the study area and are likely dependent on a variety of factors specific to each well. Comparing the Quadrant method to the commonly used Kendall's tau correlation, which requires different assumptions about aquifer behavior, the methods produced similar correlations when sample sizes were large and the time interval between samples was relatively short. Sample sizes were then artificially restricted to determine correlation reproducibility. Despite being less reproducible overall, the Quadrant method was more reproducible when there were large time intervals between samples and very small sample sizes (*n* ~ 4), but not as reproducible as significant (p ≤ 0.1) Kendall's tau correlations. Therefore, the Quadrant method may be useful for further investigating the effects of pumping in cases where Kendall's tau does not produce significant correlations.

## Graphical abstract

We introduce an interval‐scaled mean pumping rate metric and apply a Quadrant correlation method to quantify correlations between groundwater pumping rates and trace metal concentrations. Quadrant correlation of pumping and trace metal data helps particularly in cases of widely spaced sampling intervals and sample sizes less than 10.

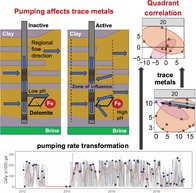

## Increased Global Groundwater Extraction Calls for Better Understanding of Aquifer Trace Metal Responses to Pumping Rates

As global population and living standards increase, global demand for fresh water also increases (Mekonnen and Hoekstra 2016), leading to the construction of deeper wells over time (Jasechko and Perrone 2021). Groundwater supplies domestic water for about half of the global population (Rodell et al. 2018). Increasingly, it has become clear that in addition to lateral transfer along permeable zones, groundwater management and pumping rate practices may cause in‐well vertical transfer of dissolved or colloidal components that influence water quality (Thaw et al. 2022). Groundwater pumping (with associated changes in aquifer conditions) clearly affects both major ion and trace element geochemistry in an array of aquifer systems (e.g., Brown et al. 2002; Ayotte et al. 2011, 2015; Xing et al. 2013; Wang et al. 2021; Bradley et al. 2024). Pumping can reduce groundwater quality, in some cases making it unsuitable for public consumption, by affecting concentrations of anthropogenic contaminants as well as naturally occurring salinity and trace metals. Understanding how trace elements such as As, B, Cr, Mn, Se, and U are affected by groundwater pumping is vital due to their effects on human health (Mitchell et al. 2011). Naturally occurring trace metal ingestion due to increased groundwater use in the twentieth century has already been linked to outbreaks of keratosis, Blackfoot disease, and various cancers (Morales et al. 2000; Mitchell et al. 2011). Thus, understanding how trace metal concentrations in groundwater respond to changing pumping conditions is critical and will continue to be so as groundwater usage, and hence pumping increases.

### The Need for Correlation Methods that Effectively Analyze Groundwater Data with Irregular Sampling Frequency

Statistical evaluation of correlations between groundwater pumping rates and various water quality parameters frequently utilizes “nonparametric” statistical methods (those that do not rely on data population means or standard deviations, for example) because sample sizes vary widely and data may not follow normal distributions. Also, groundwater data often contain many instances of measurements below detection limit or non‐detects, along with parameter units that span many orders of magnitude. In particular, rank‐based methods like Kendall's tau and Spearman's rho may provide robust correlations that can help assess the impact of pumping on groundwater constituents, but they require strict assumptions about the time interval over which to quantify the pumping variable. Spearman's rank correlation, for example, has been applied to determine relationships between depth to water and above‐detection‐limit concentrations of As and U (Landon et al. 2008). However, their approach assumes the trace metal concentration responds primarily to changes in water level for all wells in the study area through the entire history of each well, regardless of the time‐course changes in any particular well. In another case, Spearman's rank correlation was calculated between the cumulative volume of water pumped over well history and total dissolved solids (Haack and Luukkonen 2013). Due to the rank‐based approach of the Spearman's method, this approach assumes that the total dissolved solids at a given time in any well in the study area is dependent on the entire history of pumping for that well rather than the change between sequential measurements.

Kendall's tau was chosen as the comparison method for this study because it is slightly more robust against extreme points in the data (where below‐detection values lie) compared to the Spearman's rank method, but otherwise behaves very similarly (Croux and Dehon 2010; Helsel et al. 2020). Both methods need an appropriate frequency of reported pumping rates in order to determine correlations between rates of groundwater pumping and trace metal concentrations. Too short of a time interval for the pumping variable could miss perturbations that affect current trace metal concentrations, while too long of a time interval could produce an artificial correlation between a no‐longer‐applicable pumping stressor and an unrelated change (or lack of change) in trace metal concentrations. Pump and slug tests may provide some indication of how long a given well will take to re‐equilibrate to a perturbation of a certain magnitude, but such an experiment would be unfeasible to conduct at every well and cannot be extrapolated to other wells in a given region unless the aquifer is extremely homogeneous.

The Central Oklahoma Aquifer (COA) contains elevated groundwater concentrations of U, As, Cr, and Se (Christenson and Havens 1998) that may be affected by pumping rates. However, the available groundwater data violate several of the necessary conditions for accurately applying the Kendall's tau method. COA lithology is very heterogeneous, so pump or slug tests cannot be extrapolated; it contains complexly interbedded sandstones, mudstones, and siltstones which vary with depth and lateral distance (Smith et al. 2009). In addition, the rate and duration of pumping varies significantly through time in the City of Norman due to changes in the volume of groundwater required due to seasonal variations in surface water sources and usage, so even if a pump or slug test has been conducted on a well of interest, it may not accurately predict aquifer response to each specific change in pumping rate (City of Norman 2018). Another limitation to the Kendall's tau method is that sample sizes must be greater than 10 if the data set contains ties (two points sharing the same *x* or *y* values) to produce an accurate calculation, which is larger than the total number of samples in several City of Norman wells (Helsel et al. 2020).

We developed an R‐based implementation of an alternative Quadrant method for computing correlations between trace metal concentrations and pumping rates that overcomes some of these limitations. In this work, we report details of this method and its application to the wells from the COA. Several hypotheses and conceptual models have been proposed to account for how metal concentrations could respond in different ways to changes in groundwater pumping conditions in the COA; those hypotheses are detailed in Tomlinson (2021). This manuscript strictly explores the effectiveness of the Quadrant method in comparison to the Kendall's tau method, using the compiled public data and additional measurements collected by Tomlinson (2021).

## Application of the Quadrant Correlation Method to Groundwater Systems: Introducing the Interval‐Scaled Change in Mean Pumping Rate

Ideal data for studying trace metal/pumping correlations would consist of daily (or almost daily) groundwater chemistry samples prior to, during, and after some change in pumping conditions so that clear initial and post‐perturbation equilibrium trace metal concentrations could be determined for every well of interest and for perturbations of specific magnitudes, like the data from a pumping experiment in eastern Wisconsin (Ayotte et al. 2011). Changes in hydrogeologic conditions in the aquifer that lead to changes in trace metal concentrations will only occur when the specific pumping event is of large enough volume/long enough duration that the pumping perturbs equilibrium or steady‐state conditions. All three correlation methods discussed here (Spearman's rho, Kendall's Tau, and the Quadrant method) assume that the net change in trace metal concentrations is related to the net change in hydrologic conditions over some window of time. Spearman's rho and Kendall's tau have been applied with the fixed time intervals discussed above; conversely, the Quadrant method, using the mean interval pumping rate metric, assumes that this window of time is the (flexible) time between trace metal samples rather than a fixed time interval. Additionally, the Quadrant method does not require sample sizes to be greater than 10 in cases with tied data. This method has its own limitations, however, as it has been shown to be less efficient than Kendall's tau and Spearman's rho when *n* > 10 and both variables are normally distributed, meaning that it requires more samples to achieve an accuracy similar to that attained with the other methods under those conditions (Croux and Dehon 2010).

The Quadrant method, like Kendall's tau and Spearman's rho, produces a correlation metric between two variables that ranges from −1 to 1. It first entails centering the data by transforming and plotting the two variables to be compared (e.g., raw well pumping rate and trace metal concentration) as Cartesian x and y axes and setting the medians for these x, y variables to 0, 0 (Croux and Dehon 2010). The quantitative correlation metric is then calculated based on the number of transformed points in each quadrant; upper right (+x, +y), lower right (+x, −y), upper left (−x, +y), and lower left (−x, −y) with the formula below:

(1) $$\begin{array}{ll} & \text{Quadrant correation}\\ & \;=\frac{\begin{array}{l} \left(\#\;\text{of upper right}+\text{lower left points}\right)\\ \;-\left(\#\;\text{of lower right}+\text{upper left points}\right) \end{array}}{\text{total}\;\#\;\text{of points}} \end{array}$$

**Figure 1:**
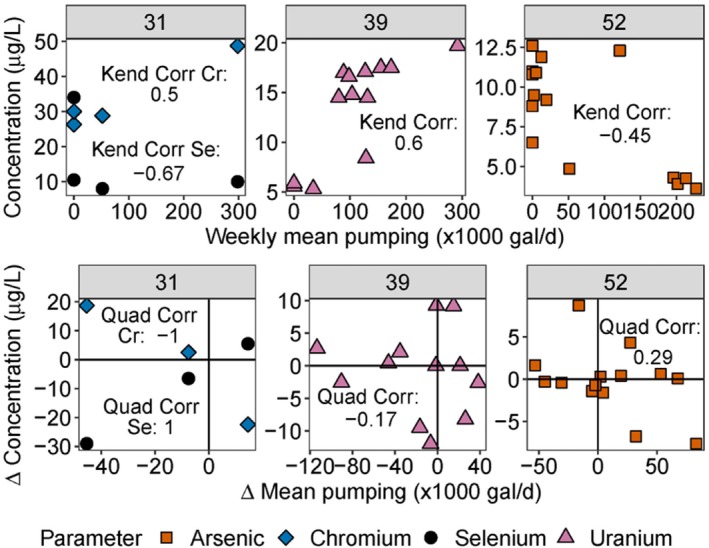
Scatter plots of three wells' Kendall's tau correlations (top) and Quadrant correlations (bottom). Each data point represents one pair of change in mean interval pumping rate versus change in trace element concentration. City of Norman well numbers withdrawing from the COA are indicated in the gray bar above each panel. Each panel includes data for one or more trace metals as indicated by the color and shape of the data point.

By modifying the centering transformation by introducing the *change in interval mean pumping rate* as the x‐axis variable, we generate a pumping metric with a flexible time window. To fulfill the centered data requirement for the Quadrant method (Croux and Dehon 2010), we converted the raw concentration and pumping rate data documented in the original data set into the change in concentration between two sequentially collected samples (as the y‐axis variable, Figure 1) and the *interval‐scaled change in mean pumping rate* between the two samples (“Δ mean pumping”, x‐axis of Figure 1). We calculated *interval‐scaled change in mean pumping rate* by taking the mean of all daily pumping values reported for the time‐period (interval) between two groundwater chemistry sample measurements, then subtracting the mean daily pumping rate calculated over the same time interval prior to the first sample (example below). This conversion meets the Quadrant method's transformation requirement by centering no change in concentration and no change in pumping rate at 0, 0. This method allows for a flexible time‐window that incorporates the available chemical data and relates the change in chemical concentrations to the change in pumping conditions over the same amount of time. However, this flexibility could also be seen as a limitation, where the period of sampling may be too long or short to not capture the water‐rock interactions of interest. We anticipate that most data sets will be limited by the availability of groundwater chemical measurements, and that, therefore, the time between measurements is sufficient to capture the relevant interactions.

Here we present an example demonstrating the process of calculating an (x, y) pair for input to the Quadrant correlation calculation. Equation 3 calculates the *change in interval mean pumping rate* between two consecutive chromium samples from Well 31 (48.7 μg/L on November 20, 2012 and 26.3 μg/L on February 23, 2017):

(2) $$\begin{array}{ll} & \frac{\sum_{11/20/2012}^{02/23/2017}\text{daily pumping rates}}{1556\;\text{days}}\\ & \;-\frac{\sum_{08/17/2008}^{11/20/2012}\text{daily pumping rates}}{1556\;\text{days}} \end{array}$$

This generates the point (14,530 gal/day, −22.4 μg/L) shown in Figure 1. In general terms, this calculation indicates that the chromium concentration in groundwater pumped from Well 31 decreased by 22.4 μg/L between November 20, 2012 and February 23, 2017 and that pumping during this time period was on average 14,530 gal/day higher than it was during the same time interval before the first sample was collected. When a sample interval contained a below‐detection limit value and an above‐detection limit value, the change in concentration was still calculated by using half of the detection limit in place of the below‐detection value, but only if the above‐detection value was above the detection limit of the other sample as well. For example, a change in concentration from <1 to 1.5 μg/L would be listed as 1 μg/L but a change in concentration from <1 to 0.8 μg/L or from <1 to <2 μg/L would be removed.

The Quadrant method then produces a correlation metric based on the instances where pumping and trace metal concentration are positively related (increasing together in the upper right quadrant or decreasing together in the lower left respectively) and the instances where pumping and trace metal concentration are negatively correlated (the upper left and lower right quadrants) as shown in the formula above. As an example, using only the data for Cr in COA Well 31 (not Se), two points fall in the upper left quadrant of the change in concentration versus change in interval mean pumping and one point falls in the lower right quadrant (Figure 1). Therefore, it has a *quadrant correlation* of −1. On the other hand, As data from COA Well 52 showed 9 points in the lower left plus upper right quadrants (increasing metal concentration with increasing pumping rate) and 5 points in the upper left/lower right quadrants, with a resulting quadrant correlation of 0.29.

(3) $$0.29=\frac{\begin{array}{l} \left(9\;\text{positive correlation points}\right)\\ \;-\left(5\;\text{negative correlation points}\right) \end{array}}{14\;\text{total points}}$$

## Application of the Method to the COA

We tested both Kendall's Tau and Quadrant correlations on available real world data for the City of Norman well field to compare the theoretical advantages and disadvantages of each method.

We used R, an open‐source statistics software and programming language (R Core Team 2019), to compile and organize four different datasets which allowed us to calculate the trends between trace metal concentrations and daily pumping rates around Norman, OK after assembling the most possible data (Appendix S1). Once combined, the raw data sets yielded 1143 sample points that included major groundwater chemical parameters, trace metal concentrations (U, As, Cr, and Se), and daily pumping information for each well. Fifty‐eight of these points were removed as erroneous duplicates and 64 were replaced with daily median concentrations in wells that were sampled more than once in a day for a given trace metal (Appendix S2). This yielded a final dataset of 1021 individual observations across 4 trace metals of interest (As, Se, Cr, and U), 156 of which were below detection, across 46 individual wells.

A pump test previously conducted in a production well and a series of surrounding monitoring wells at variable depths near Norman, OK showed that the aquifer water level only responded to pumping when the monitoring well was screened at the same depth as the pumping well, and that the water level returned to pre‐pumping levels approximately 8 days after pumping ceased (Mashburn et al. 2018). Therefore, we calculated the Kendall's tau correlation between the mean daily pumping rate (volume per 24‐h period) over the 8 d prior to (and including) the sample collection date and the sample concentration for each trace metal and well (Figure S1). We used mean rather than median daily pumping rate because the water level may be sensitive to large pumping rates. These large values would be missed by using the median due to the large number of zero or low pumping values present in the dataset. The NADA R package (Lee 2020) incorporated below‐detection values in the Kendall's tau correlation by counting changes from below detection to values above the same detection limit as increases and changes from values reported above the detection limit to below detection as decreases.

**Figure 2:**
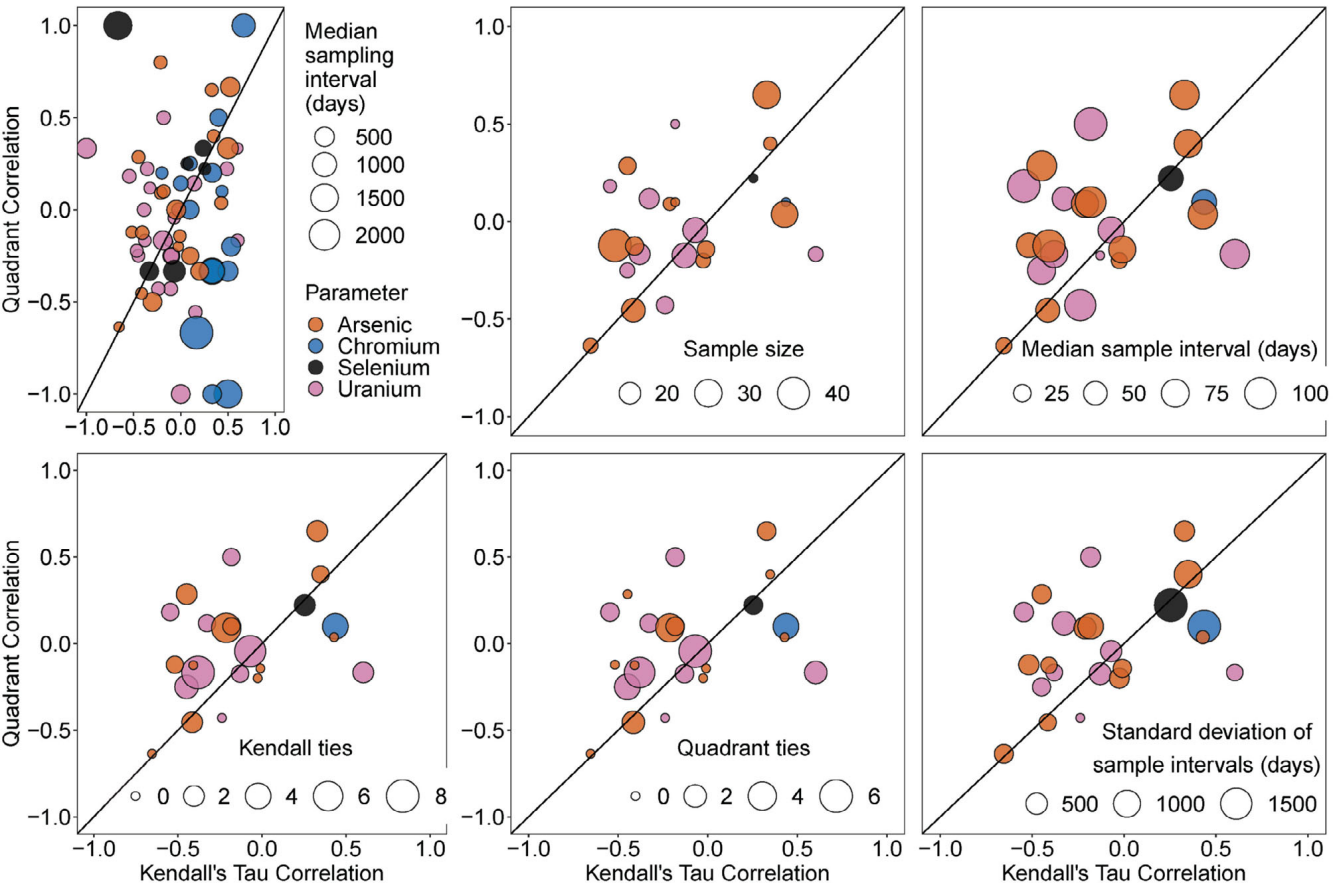
Kendall's tau correlations vs. Quadrant correlations for all wells and parameters where enough data existed to compare. Each data point corresponds to a single trace metal/ well correlation calculation, for example as shown in each panel of Figure 1. The “sample size” reflects how many points were available for the correlation calculation (how many points on each panel of Figure 1). The upper left panel shows all wells and parameters while the other panels only show correlations with sample sizes greater than 10, which tend to be much more similar. The rest of the panels (*n* > 10) have different parameters for dot size (sample size, median sample interval in days between samples, number of ties in each method, and standard deviation of sample interval (in days). Calculated R^2^ values, forcing *x* = *y*, are −0.68 and −0.58 for the full dataset in the upper left panel and all other panels, respectively.

Considering the entire dataset, 85 sample sets of As, Se, U, and Cr concentrations across 31 wells yielded at least 4 time‐points which could be theoretically used to generate both a Quadrant and Kendall's tau correlation, assuming no ties were present. The initial Quadrant method produced 67 correlations across 28 wells once the tied points were factored in. Kendall's tau produced 45 correlations across 25 wells that met its use conditions of having no ties or sample sizes greater than 10. A merged dataset that contained 63 correlations across 27 wells with both a quadrant correlation and a Kendall's tau correlation (including Kendall's tau correlations that could still be generated despite having ties in sample sizes below 10) were then used for further comparison. We plotted Kendall's tau coefficients against our calculated Quadrant correlations for each well and trace metal to determine how similarly they behave across an array of conditions using the COA dataset (Figure 2).

Furthermore, to test the effect of small sample sizes on both correlation metrics, we used the base‐R *sample* function (seed = 1) to randomly generate subsets of the original data for each trace metal and well (R Core Team 2019). The size of each subset was randomly chosen between four and 10 points from the original data before transformation (four being the minimum number of points that can produce a Kendall's tau correlation and a Quadrant correlation). The samples were randomly chosen for each subset (using the *sample* function with seed = 2), so an eight‐point data subset for As in well 52, for example, could contain any set of 8 samples from the original data regardless of date (R Core Team 2019). Instances where the randomly generated number of samples between four and 10 were greater than the number of samples available were excluded from the comparison; 35 randomly generated correlations were compared across 21 wells.

## Results and Discussion

Trace metal—pumping trends varied significantly when comparing individual trace metal concentrations across different wells and when comparing different trace metal concentrations within the same well (shown graphically and spatially for reference in Figures S2‐S7). Sufficient data was available for only one or two of the four studied trace metals within a majority of the wells (Figure S2), limiting the applicability of the models to test mechanistic trends and hypotheses for each trace element.

In the COA, geogenic trace metals generally result from water‐rock interactions in deep, older waters with ages in the thousands of years (Parkhurst et al. 1996). As a starting framework to develop testable ideas linking trace‐metal concentration and pumping rates, Tomlinson (2021) hypothesized that lower (circum‐neutral) pH water would flow from upper screened/perforated aquifer zones into deeper (more alkaline) zones along the well bores during inactive periods soon after a well was turned off, leading to sorption and a subsequent decrease in certain trace metal concentrations such as arsenate, chromate, selenate, and uranyl. However, during pumping periods, the pH should rise causing desorption and increasing concentrations of these trace metals. In general, calculated correlations supported these hypotheses; however, the correlations for each well‐metal combination were shown to depend on specific factors related to characteristics of the aquifer (e.g., percent sand), pumping (frequency on/off and volume of water), well (location east/west, depth, number, and distribution of screened/perforated intervals), and speciation of the trace metals (e.g., Ca‐carbonate ions in solution uniquely mobilize U), which likely lead to the observed variability in data from the COA (Tomlinson 2021). While the analyses proved inconclusive, this manuscript is focused on constraining conditions for the appropriate use of the Kendall's tau and Quadrant methods.

In comparing the two methods of calculating metal‐pumping correlations, neither the Kendall's tau method nor the Quadrant method produced consistent correlations between trace metals and pumping conditions that could be applied across the entire field of 41 wells. We observed different signs (inconsistent results) between the Kendall's correlation and the Quadrant correlation in 22 of the 63 correlations comprising the full set of trace metal and pumping data, spanning 16 out of 27 wells.

Specifically, the data transformation used in the Quadrant method accounts for some of this inconsistency by sometimes producing trends that are opposite from the raw data plotted in the Kendall's tau method due to the use of the *interval‐scaled change in mean pumping rate* parameter, such as for Cr in well 31 (Figure 1, left, diamonds) which was assigned a negative (−1) Quadrant correlation metric but a positive Kendall's tau correlation (+0.33, “calculated_parameters_full.xlsx” in Supporting Information). In cases where there are clear differences in trend quality between the raw and transformed data, cross plots comparing both methods indicate which one is likely to be more representative of the data. For example, the raw data generates a visibly stronger relationship in well 39 for U than the transformed data, so a positive correlation between pumping and trace metals is more likely (Figure 1, center). Transformed data generates a stronger relationship in Well 31 for Se, again making a positive correlation more likely (Figure 1, left, circles). Other correlations did not show such a clear difference in method effectiveness, such as Cr in Well 31 and As in Well 52 (Figure 1). Visual trends may be misleading, however; for example, As in well 52 generated a positive quadrant correlation despite showing a visibly negative trend (Figure 1, right) because there were more points in the upper right and lower left quadrants even though they tended to be closer to 0.

Sample size and median sampling interval both had an influence on how similarly the two methods performed, while variability in the sampling intervals (determined based on the standard deviation) did not. The methods behaved most similarly when sample size was greater than 10 and the median sampling interval was relatively small; further increases in sample size beyond 10 did not increase method similarity (Figure 2, Table S1). In our dataset of 63 correlations, only 23 had a sample size of 10 or greater, including 12 for As, 9 for U, 1 for Cr, and 1 for Se (Figure 2). Kendall correlations with fewer ties (multiple points with the same x or y value, representing either no change in pumping rate or no change in concentration) tended to behave more similarly to their respective Quadrant correlations than those with more ties, but the number of ties in the Quadrant correlations (corresponding to either the *interval‐scaled change in mean pumping rate* or the change in concentration values equal to zero) had no clear influence on how similarly they performed to the Kendall correlations (Figure 2). The quadrant method also produced fewer ties overall, with a median of 0 and a range of 0 to 6 ties per correlation. The Kendall method had a median of 1 and a range of 0 to 8 ties per correlation (Table S1).

Kendall's tau correlations and Quadrant correlations were then calculated from 35 random subsamples of the original data collected from each individual well; the signs of the coefficients were compared to those generated from the full dataset for each well. The correlations for a given well and parameter were “consistent” if the sign on the subset correlation matched the sign on the full data set correlation. Four of the 35 small sample Kendall's correlations were inconsistent with their respective full sample Kendall's correlations, while five of the 35 small sample Quadrant correlations were inconsistent with their respective full sample Quadrant correlation results. The inconsistent Kendall's correlations tended to have the smallest sample sizes (*n* ~ 4) and larger median sample intervals; however, this trend was not observed in the Quadrant correlations (Table 1). Both sets of inconsistent correlations tended to have larger sample interval standard deviations than the sample sets with consistent correlations. This trend was stronger among the inconsistent Quadrant correlations, which had a much larger range of sample interval standard deviations than the inconsistent Kendall's correlations (Table 1). Unexpectedly, the average number of points was higher in the inconsistent Quadrant correlations (median = 8) than the consistent ones (median = 6), in contrast to the behavior with the Kendall's tau correlations (7 and 4, respectively, Table S1). None of the inconsistent Kendall's correlations were significant at the α = 0.1 significance level. The number of ties in the inconsistent correlations was not significantly different than the number of ties in the consistent correlations for either method (Table S1).

**Table 1:** Sample Statistics (Minimum, Median, and Maximum Values) for Kendall's Coefficients and Quadrant Correlations that Changed Signs (Inconsistent) or Kept the Same Sign (Consistent) Between the Randomly Generated Subset and the Original Data Set

	Kendall Correlations Consistent (31)			Kendall Correlations Inconsistent (4)			Quadrant Correlations Consistent (30)			Quadrant Correlations Inconsistent (5)		
	*n*	Sample interval med (d)	Sample interval SD (d)	*n*	Sample interval med (d)	Sample interval SD (d)	*n*	Sample interval med (d)	Sample interval SD (d)	*n*	Sample interval med (d)	Sample interval SD (d)
Min	4	22.5	47.75	4	110	384.5	4	22.5	47.75	5	75	91.1
Med	7	178	512.05	4	353.8	633.5	6	192.2	505.27	8	171	636.3
Max	10	1354	1779.2	9	792	872.8	10	1354	1779.2	10	322.5	1315.8

Note: n = number of points in the randomly generated subsets. Sample interval = number of days between subsequent samples; both the median (med) and standard deviation (SD) statistics are shown.

This comparison between the Kendall's tau and the Quadrant method shows that the two methods behave most similarly when the sample size is greater than 10 with small median sample intervals. It also demonstrates that the Kendall's tau behaves more similarly to the Quadrant method when the sample set contains fewer ties in the raw data. Kendall's tau was least reproducible with subsample sizes *n* ≤ 10 for datasets with large median sample intervals and the smallest possible sample sizes (*n* ~ 4), while the Quadrant method was less impacted by these constraints. This is a logical byproduct of the differences in computation approaches employed by these methods, as large sample intervals strongly deviate from the week‐long sampling window used in the Kendall's tau method. The Kendall's tau method generated slightly fewer inconsistent results with reduced data, so despite the theoretical limitations of the Kendall's tau, it still performs similarly or better than the quadrant method for data sets with fewer than 10 points. However, the quadrant plot may be preferred when the data are widely spaced apart (high median sample interval) and/or when sample sizes are close to four since those characteristics were more strongly associated with the inconsistent Kendall's tau coefficients.

## Conclusions

The *interval‐scaled mean pumping rate* provides a mechanism to analyze data where water flows may turn on and off over a wide range of time scales and volumes. The Quadrant method may be more likely to produce robust correlations between trace metal concentrations and well pumping rates than the Kendall's tau method if the following conditions are met:

1. Aquifer response time to pumping is unknown or too variable to predict across the study region.
2. A change in concentration vs. *interval‐scaled change in mean pump rate* cross plot shows an equally strong or stronger relationship between variables than a concentration vs. mean weekly pump rate (or other appropriate time interval) cross plot.
3. The samples in the data set were collected at widely spaced time intervals.
4. Sample size is less than 10.
5. Kendall's tau produces insignificant results (p > 0.1).

Under the above conditions, the Quadrant method can be used to quantify pumping/trace metal relationships across an entire range of historically collected data with a lower likelihood of error than the Kendall's tau method. This can help municipalities drawing water from heterogeneous aquifers evaluate how increased magnitude or frequency of pumping could affect their water quality, even if the data are not collected frequently. We recommend future studies evaluate correlations between pumping and trace metal concentrations by performing experiments to collect data for both at a range of time scales—hours, days, weeks, etc. to better understand the water‐use practices that influence water quality.

Although our study investigated connections between pumping and water quality, the Quadrant method can likely also be used for other types of data with infrequent data collection. The transformation of data into interval‐scaled mean values, such as the interval‐scaled mean pumping rate from this study, could be used for other types of hydrologic data and for other purposes than investigating trace metals. For example, hypotheses related to the influence of climate on groundwater water quality could be explored through the relationships between recharge estimates and shallow groundwater characteristics (Whittemore et al. 1989), or many other parameters. We hope the community of researchers can adapt the concepts from this work and the code found in the Supporting Information to advance our understanding of groundwater processes. Additionally, we recommend that deliberate studies be performed to test the link between pumping rates and trace metals in a range of aquifer/well types that investigate hypotheses related to multiple screened intervals/depths, pumping frequency and volumes, aquifer characteristics, etc. to generate robust predictions and advice for managing well usage that can be widely applied.

## Authors' Note

The authors do not have any conflicts of interest or financial disclosures to report.

## Supporting information


**Appendix S1.** Data collection, management, and formatting.
**Appendix S2.** Procedure for reducing the original dataset.
**Figure S1.** Process for computing the weekly mean pumping rate for each sample taken from the well.
**Figure S2.** Transformed trace metal‐pumping trends for all Norman wells.
**Figure S3.** Raw trace metal data with pumping across the Norman wellfield.
**Figure S4.** Arsenic correlation results map.
**Figure S5.** Chromium correlation results map.
**Figure S6.** Selenium correlation results map.
**Figure S7.** Uranium correlation results map.
**Table S1.** All parameters in randomized subset comparison.

**Data S1.** Downloadable file including the full source code for the Quadrant correlation method and raw data files used to create the plots shown in the manuscript.

## Data Availability

The data that support the findings of this study are included in the Supporting Information associated with this manuscript.
