# Supplementary material for: Applying the Quadrant Method for Pumping‐Trace Metal Correlations in Variable Time, Low‐Data Systems
Source: Ground Water. 2024 Dec 20;63(2):256–64. doi: 10.1111/gwat.13458 (PMC11875045; doi:10.1111/gwat.13458)
Supplement: Supplementary file 1 — Appendix S1. Data collection, management, and formatting. Appendix S2. Procedure for reducing the original dataset. Figure S1. Process for computing the weekly mean pumping rate for each sample taken from the well. Figure S2. Transformed trace metal‐pumping trends for all Norman wells. Figure S3. Raw trace metal data with pumping across the Norman wellfield. Figure S4. Arsenic correlation results map. Figure S5. Chromium correlation results map. Figure S6. Selenium correlation results map. Figure S7. Uranium correlation results map. Table S1. All parameters in randomized subset comparison. [file GWAT-63-256-s001.pdf]

Supporting information for:

Applying the Quadrant method for pumping-trace metal correlations in variable time, low-data systems

Zachary D. Tomlinson, Kato T. Dee, Megan E. Elwood Madden, Andrew S. Elwood Madden

Appendix S1: Data collection, management, and formatting

A total of 4 unique datasets were combined for the data used in this study. These include: (1) new data from samples collected in the field by this research team (Tomlinson 2021), (2) compliance data recording concentrations of chemicals with EPA-mandated safe minimum concentrations (collected by the City of Norman), (3) National Water Information System (NWIS) data stored by the USGS for select Norman wells, and (4) pumping data showing how much water was pumped from each well each day (U.S. Geological Survey 2016). Each dataset required different types of pre-processing and standardization before the data from these different sources could be combined and utilized as a single dataset.

Norman EPA compliance data

Norman's EPA compliance data was originally formatted in a list of individual parameters and results. First, confidence intervals were removed from the data and values below detection limits were replaced with the detection limit as the result value by removing the < symbol. An additional column was then added to the data with the value "TRUE" if the concentration fell below detection limits. This additional column was later used in the Kendall's tau correlation coefficient calculations.

Subsetting the compliance data to parameters of interest for the study also took several steps. Since each parameter was input as a table entry rather than a column label, there was a lot of variation in the entries themselves that needed to be standardized. Arsenic measurements, for example, were labeled as "Arsenic, total" and ARSENIC in the dataset. Several of the entries also had extra spaces, which R reads as unique parameters. In order to standardize the parameter names, the *grepl* function in R was used to search for any parameters that included the uppercase and lowercase (except for the first letter) versions of the parameters (R Core Team 2019). The key reason for using this instead of other subsetting functions was the include feature, which selects parameters that are not exact matches to the search parameters if the name of the search parameter is somewhere in the character string. For example. the *grepl* function treats "Arsenic, total" and "Arsenic" the same if searching for the string "Arsenic". A snippet of the code used to do this is shown below:

```
data_subset = data[grepl('Arsenic|ARSENIC|. . .|last_parameter' , data$parameter) , ]
```

Essentially, the code above searches for any character strings in data frame "data" that contain the sequence "Arsenic" or "ARSENIC" in the parameter column and returns those rows in the data_subset (the "|" symbol is the logical operator OR in R). This way, the code can capture any combination of parameter names as long as they are spelled correctly and the first letter is

capitalized. Adding the additional code line "unique(data$parameter)" can determine if the two conditions above are met. This data met those conditions; therefore, no additional character terms were needed.

Once the data subset was produced from all of the parameters of interest to this study, the variable parameter names were replaced with consistent ones by using the *grepl* function to generate indices of equivalent parameter names ("Arsenic" and "ARSENIC" for example) and then replacing the characters with a unified name. In addition to the parameter names, the sources (well names) and units were unified as well. R function *str_remove_all* in the *tidyverse* package (Wickham et al. 2019) removed "well" and "WELL" characters in front of the numbers that the City of Norman used to label their wells. Lastly, the *grepl* function was again used (along with the *which* function) to find rows where arsenic, selenium, chromium and uranium concentrations were reported in mg/l (instead of the more widespread µg/l). Once the row numbers were determined, it was only necessary to multiply the results by 1000 and change the unit name.

NWIS Data

The R package *dataRetrieval* (specifically the function *readNWISqw*) was used to read data directly from the U.S. Geological Survey National Water Information System (NWIS) database into R (De Cicco et al. 2018). Only arsenic, selenium, chromium, uranium, and depth parameter codes were called; codes 01000, 22703, 01030, 01145, and 00003, respectively (U.S. Geological Survey 2016). In addition to parameters, the function also needed a list of wells to call. The USGS published a list of City of Norman wells with their corresponding USGS well names in a 2009 report; this well list was used to determine well site numbers for the function call (Smith et al. 2009). Like the EPA compliance data above, the dataset produced from this R function was formatted as a list of individual observations and similar processing methods were required. NWIS data contains a remark column with a "<" value in every row that contains a value below detection limit (U.S. Geological Survey 2016); this was easily converted into values of "TRUE" to be used in the later calculations. Unlike the previous data, each parameter was listed as its respective code, so there was no need to make the names uniform. Code names were replaced with the parameters they represented, USGS well names were replaced with City of Norman well names, units were added based on parameter code descriptions, and the data was added to the other datasets with the R function *rbind*.

All of the other data sources consist of well-head measurements, while NWIS data contains both well-head measurements and depth-specific measurements. Depth-specific data needed to be filtered out of the combined Norman dataset because shallow depth-specific measurements do not show the variations in trace metals seen in deeper samples. Likewise, deeper samples could overemphasize certain trends. Keeping the depth-specific measurements in this dataset could therefore obscure some of the trends. There are four steps to filter out these types of data from an NWIS data call:

1. Add parameter 00003 to the *readNWISqw* function to pull depth information from NWIS (De Cicco et al. 2018).
2. Make a subset of data containing only parameter 00003 and the columns date, time, source (well name), and result with *subset* and/or *select* and *filter*.
3. Change the name of the result column to something else, like "Depth".
4. Join this subset to the original dataset by date, time, and source.

Assuming that all samples have a unique combination of date, time, and source, the four steps above will generate an extra column that easily distinguishes the depth-specific data from the other data. There were a number of samples with repeat date, time, source, and parameter values which needed to be aggregated (75 total observations across all parameters). Some duplicated samples were clearly corrections to typos; for example, arsenic entries with values of 1500 and 1.5 µg/L. Others were less obvious, like alkalinity measurements of 335 and 340 mg/L $CaCO_3$ reported for the same date and time, for example. For duplicate entries with differences greater than 10 units, the minimum value was taken to ensure that decimal point corrections were used in place of the original values. Duplicate entries with differences less than 10 were averaged together. Although duplicate samples (not intended for analysis) are collected regularly with environmental data for quality assurance (QA) purposes, they are not a likely major contributor in the duplicate data here. The QA data would have to be misreported as regular field data to get mixed in with the collected data. Even if some QA data did get misreported, there was no way to differentiate QA data from regular field data with the published information, so averaging repeat measurements halved the error that would come from using a duplicate sample in place of the regular field sample.

Duplicate entries with arsenic concentrations of < 1 and 1.2 µg/L were be averaged together, for example, so there also had to be a rule for how to report samples with at least one below detection value. If the sample(s) reported as not below detection had a greater concentration than the sample(s) reported as below detection, not below detection was listed and only above-detection concentrations were used. If the samples reported as below detection had an equal or greater value to the samples reported as not below detection, below detection was listed. Note that this only occurred for differences of less than 10 µg/L or mg/L.

Personally collected field data

15 City of Norman wells with historical data were sampled from the well head during March and April 2021 in order to provide additional time-points for pumping-chemistry comparisons. Tubing was connected to the sample port nearest to each well, which fed the sample water into the bottom of a bucket which was tilted slightly to allow flow along one section of the lip; this served as a basic flow-through cell. A multi-parameter probe continually collected pH, oxidation-reduction potential (ORP), dissolved oxygen (DO), conductivity, total dissolved solids (TDS), temperature, and pressure data from the bottom of the bucket near the inlet, which limited interference from the atmosphere. The flow-through cell was also left out of the sun whenever possible to preserve temperature accuracy. Once pH, conductivity, and TDS stabilized, four

readings (one every two minutes) were collected for each field parameter. Then, we filled sample jars for metal (500 mL), anion (100 mL), and organic matter (1 L) analysis, stored them with ice, and turned the water off. The probe stayed in the flow-through cell for at least twenty additional minutes at each site without water running to get a more accurate DO measurement, because DO consistently dropped while the other measurements stabilized. Most of the wells had very low turbidity and were not filtered before being put in the sample jars; one well had visible colloids and was filtered through a 0.45 μm filter before the metal and anion collection. An Erlenmeyer flask was also filled with 100 mL of water (using a graduated cylinder for accuracy). The Hach Alkalinity Test Kit, Model AL-DT was used (along with its outlined procedure) with the Erlenmeyer flask to determine alkalinity from titration. Back at the lab, metal samples were acidified with 1 mL of 15 M nitric acid. The Atomic Spectroscopy Lab at Colorado School of Mines analyzed major and trace metal concentrations with ICP-AES and major anion concentrations with IC.

For quality control, the multi-parameter probe was calibrated three times over the course of the field work. No major corrections were needed, suggesting the instrument remained consistently calibrated during the field work. Additionally, anything that was in contact with the sampled water was rinsed with native water at least three times prior to storage (including the bucket, all sample containers, the Erlenmeyer flask, and the graduated cylinder). The bucket, Erlenmeyer flask, graduated cylinder, and multi-parameter probe were also rinsed with DI water and air dried after each field day. Results were added to the other data sources with formatting similar to the Norman EPA compliance data. All field parameters (other than DO, which was reported as its 5th measurement after the water stopped flowing) were reported as averages of the four readings collected once the readings stabilized.

Pumping data and aggregate pumping metrics

The City of Norman keeps a record of the volume of water taken from each well on each day. A for loop in R iterated through each sample record and computed the mean daily volume of water extracted over each week leading up to the sample day (including the sample day, so 8 total days). Some days were missing from the record, so instead of using the *mean* function, the *sum* function was used divided by 8 total days to get the mean, which assumes that the well was not pumped on the day with missing data (Fig. S1). The same for loop computed the number of days on (pumping volume > 0) for each weekly period. Then, using the *left_join* function in the *dplyr* R package (Wickham et al. 2021), the pumping information was joined to the existing sample data discussed above by the date and well ID parameters. Thus, each day on which a sample was collected from any given well was linked to a specific volume of water extracted and the pumping conditions on the week prior to the sample being extracted.

Figure S1: Process for computing the weekly mean pumping rate for each sample taken from the well

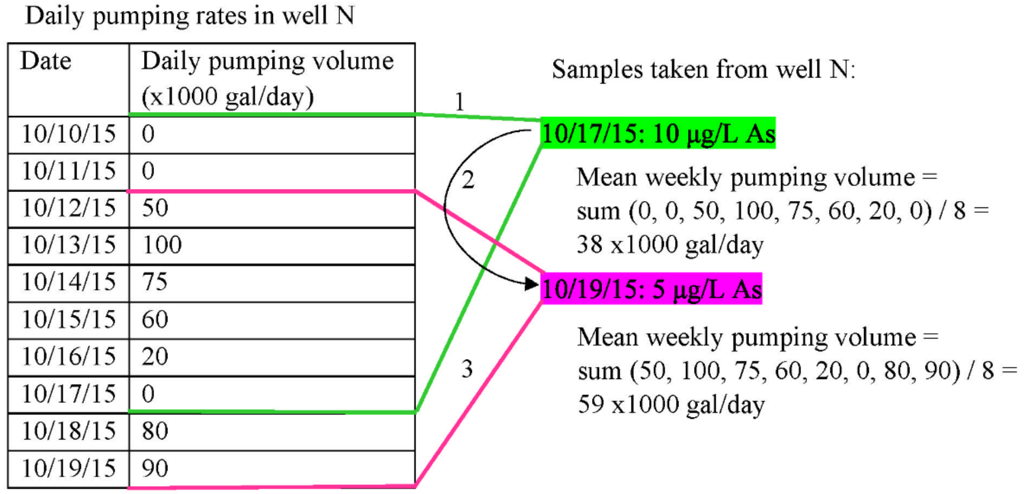

Process for computing the weekly mean pumping rate for each sample taken from the well. In step 1, the code looks at the well's daily pumping volumes starting 8 days before the well was sampled and ending on the day that the well was sampled, then computes the mean pumping rate. In step 2, the code iterates to the next sample. Step 3 is a repeat of step 1 except the sample date is different, so the 8-day daily pumping window produces a different mean weekly pumping volume.

Appendix S2: Procedure for reducing the original dataset

"Duplicate" values are defined as sample values assigned to the same date, time, well, and parameter from the combined dataset above. The original combined dataset contained 58 sets of duplicate values. Some of these points were clear corrections to misplaced decimals (such as duplicate arsenic concentrations of 2.6 and 2630 µg/L), so duplicate measurements with uncharacteristically high concentrations of 500 µg/L or higher were filtered out. Other duplicate measurements had conflicting detection limit values, like selenium concentrations of < 10 and 2.7 µg/L. In these cases, the higher value was used (< 10 µg/L). Lastly, we averaged together duplicate measurements that were sufficiently close (less than 10 units) with no way to differentiate them. Although averaging sets of duplicate measurements is not ideal when more information can be obtained, it does halve the error that would occur from selecting the wrong measurement. Other wells had multiple samples taken from a given well on the same day, even though their sample times were unique. These would be tied to the same pumping information since only one volume per well per day was reported. Therefore, the median concentration was taken for multiple measurements of the same parameter in the same well on the same day, taking the final dataset for correlation analysis down to 1021 trace metal measurements.

Figure S2: Transformed trace metal-pumping trends for all Norman wells

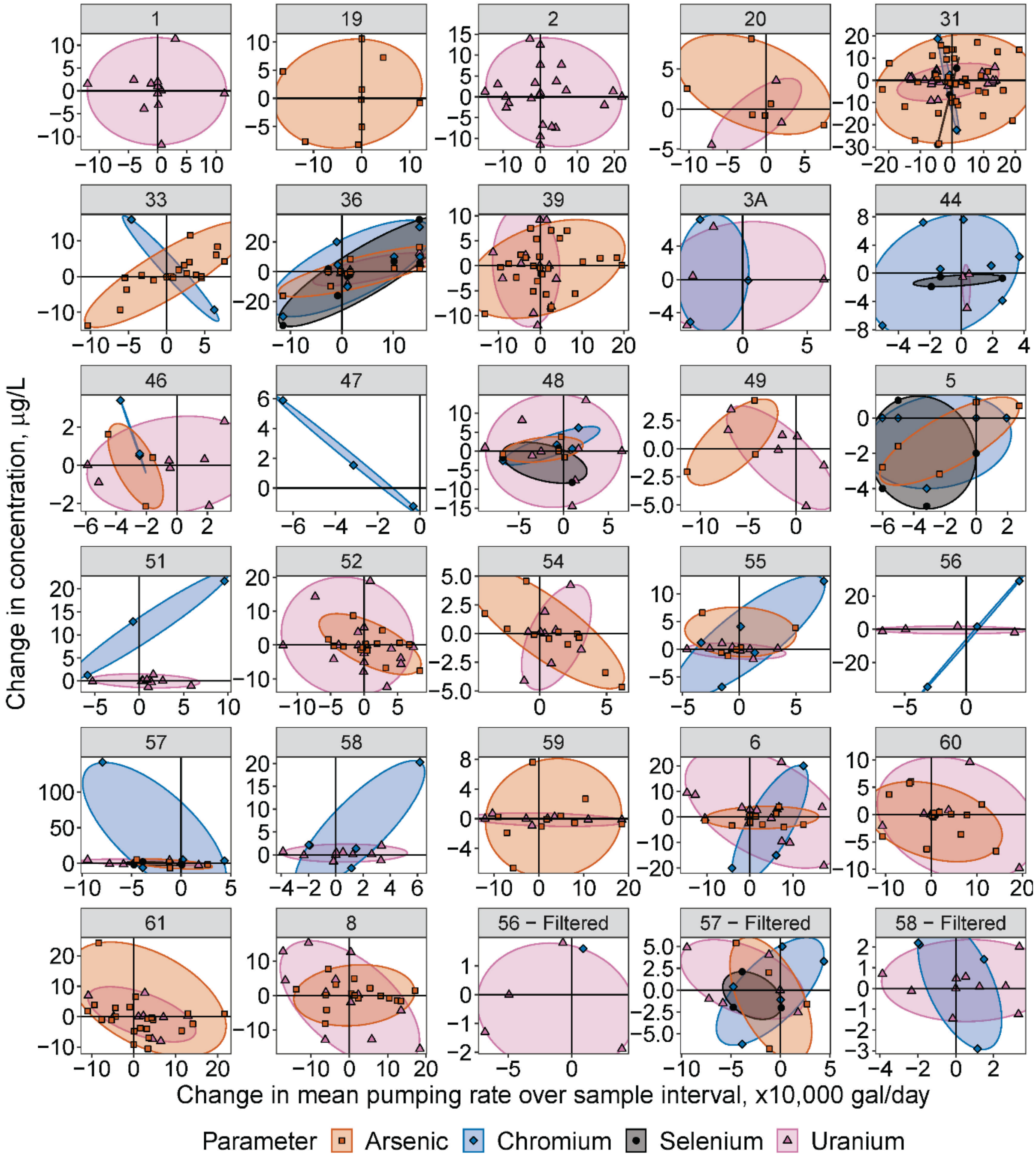

Trace metal trends with pumping across the Norman wellfield. City of Norman well numbers are indicated in the gray bar above each panel. Each point represents a set of consecutive samples for a particular trace metal. The x value for each point is the change in interval mean daily pumping rate (x10,000 gal/day). The y value for each point is the change in trace metal concentration from the earlier sample to the later sample. Ellipses mark the extent that all points for that trace metal fall within and therefore do not correspond to a particular statistic. Wells 56, 57, and 58 also include zoomed-in panels.

Figure S3: Raw trace metal data with pumping across the Norman wellfield

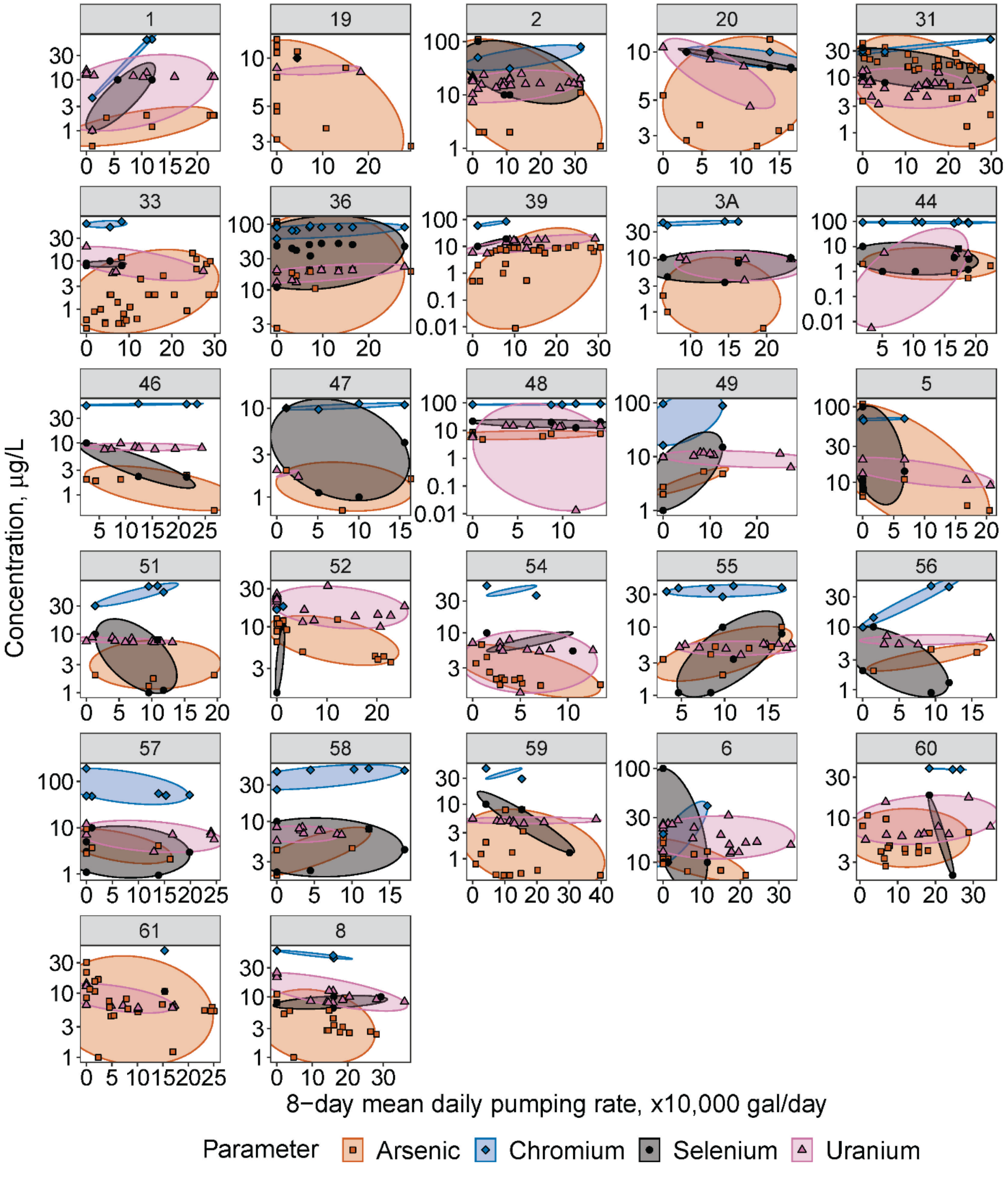

Raw trace metal data with pumping across the Norman wellfield. City of Norman well numbers are indicated in the gray bar above each panel. The x value for each point is the mean daily pumping rate over the week preceding each sample (x10,000 gal/day). The y value for each point is raw trace metal concentration at that sample time. Ellipses mark the extent that all points for that trace metal fall within and therefore do not correspond to a particular statistic.

Figure S4: Arsenic correlation results map

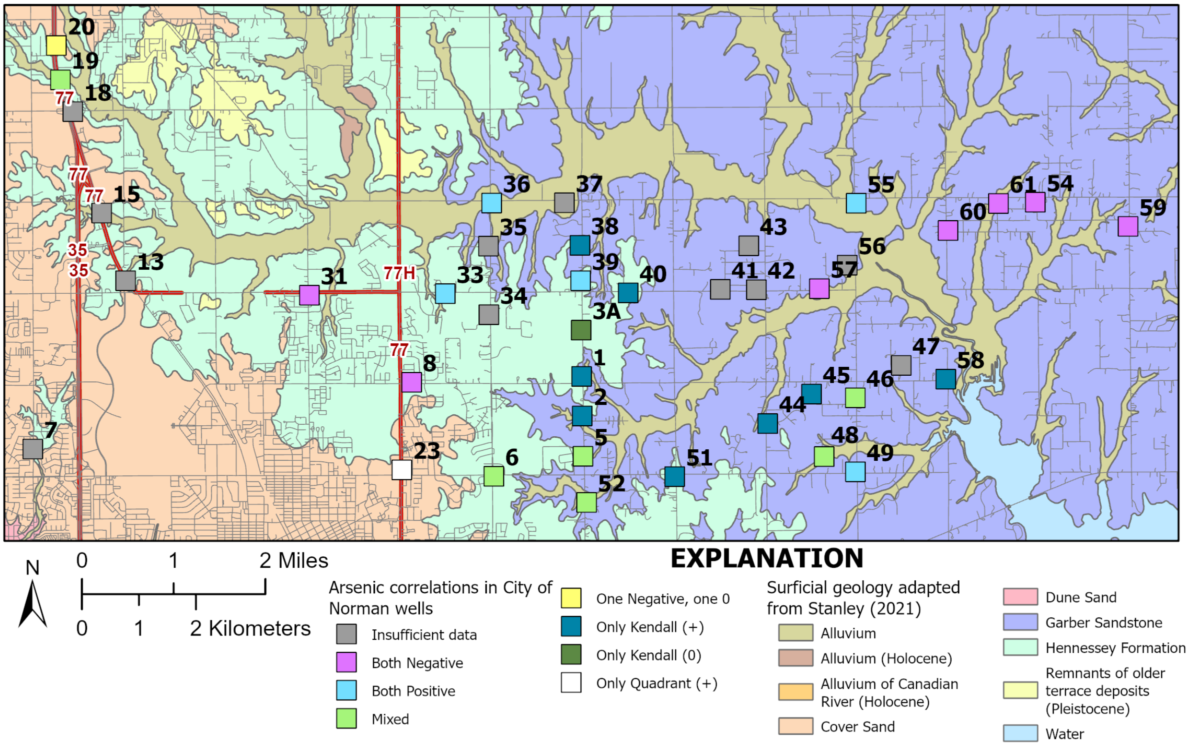

Figure S5: Chromium correlation results map

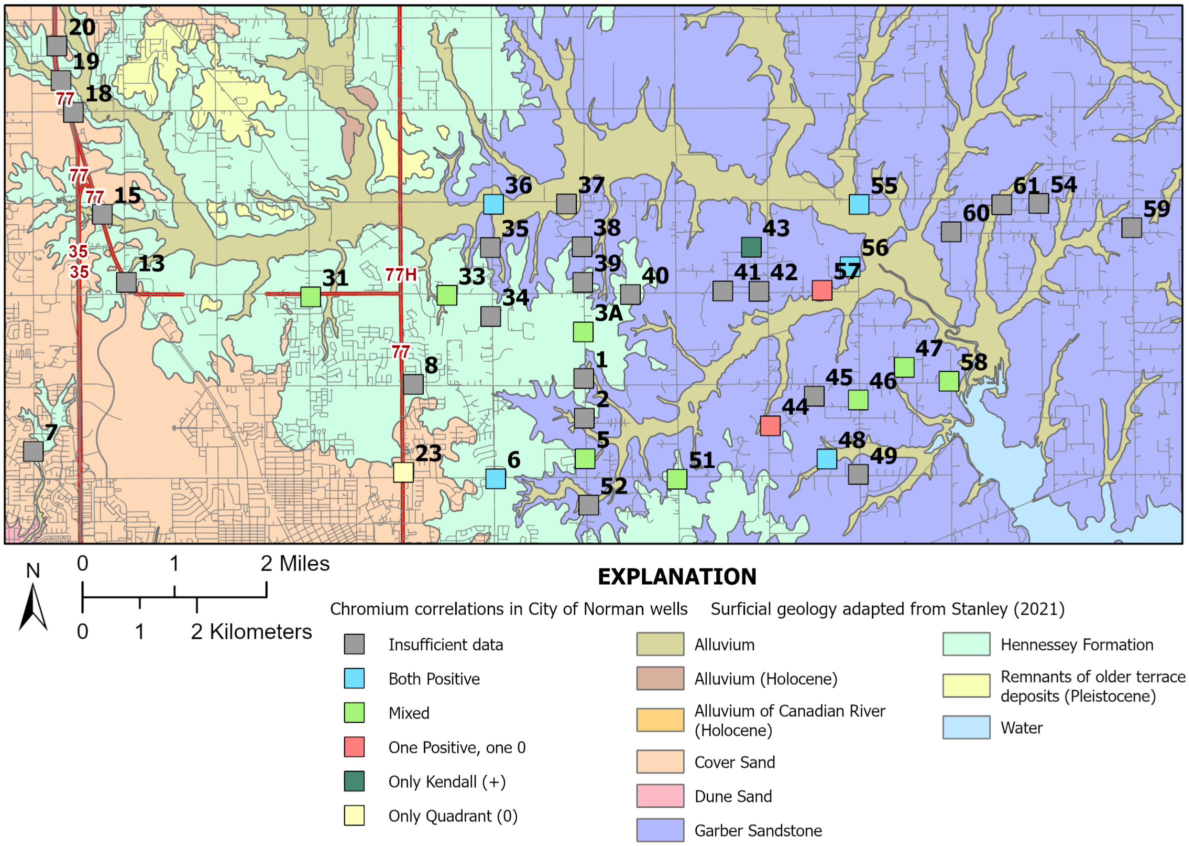

Figure S6: Selenium correlation results map

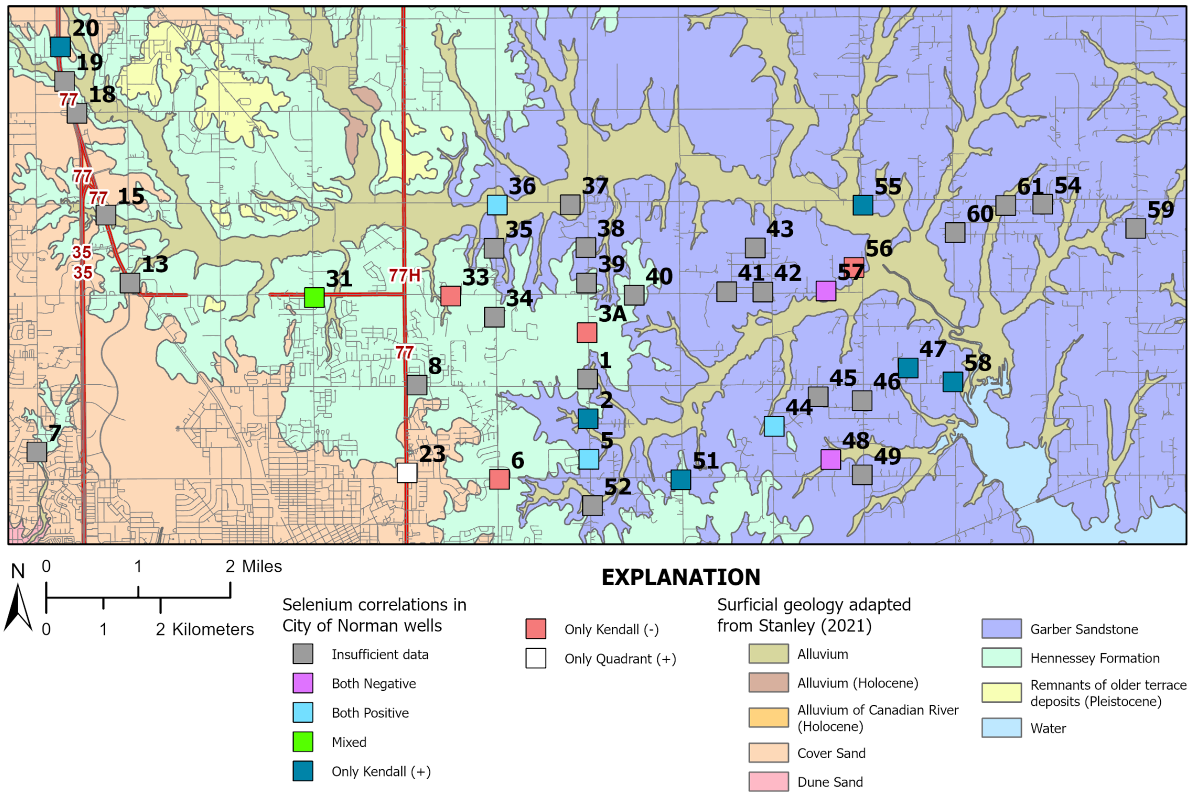

Figure S7: Uranium correlation results map

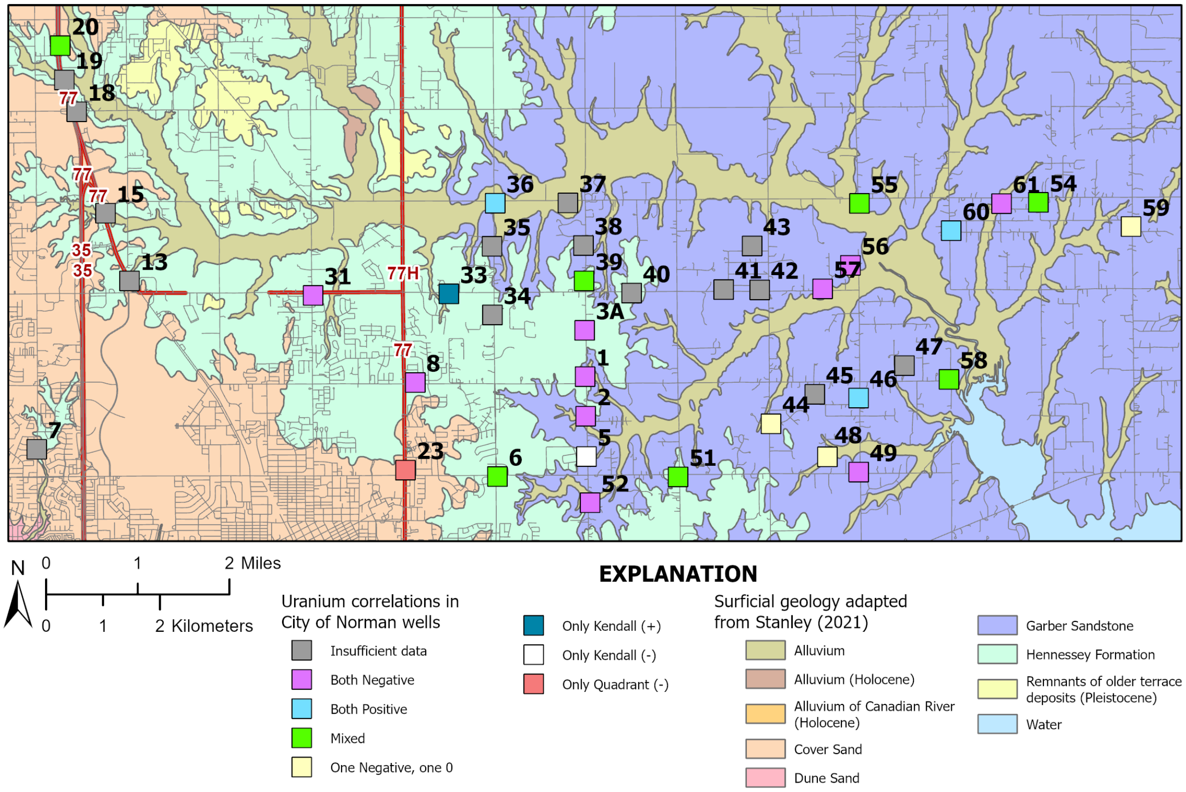

Table S1: All parameters in randomized subset comparison

	Kendall's tau consistent (31)						Quad consistent (30)				
	Ties	Median sample interval (days)	Sd sample interval (days)	p value	n	n rem-oved	Ties	Median sample interval (days)	Sd sample interval (days)	n	n rem-oved
Min	0	22.5	47.8	0.0187	4	1	0	22.5	47.8	4	1
1st Quad	0	83.5	319.6	0.1010	5	2	0	101.8	341.6	4	2
Median	0	178	512.1	0.3414	7	5	0	192.2	505.3	6	4.5
Mean	0.7	266.4	620.6	0.4155	6.9	7.7	0.3	300.4	622.7	6.5	7.1
3rd Quad	1	313.8	711.2	0.6721	9	11	0	347.4	791.3	8.8	8.5
Max	5	1354	1779.2	1	10	34	3	1354	1779.2	10	34
	Kendall inconsistent (4)						Quad inconsistent (5)				
Min	0	110	384.5	0.3082	4	3	0	75	91.1	5	3
1st Quad	0	152.4	440.4	0.3554	4	3.75	0	92	357.2	6	4
Median	1	353.8	633.5	0.644	4	5	0	171	636.3	8	6
Mean	1	402.4	631.1	0.6491	5.3	6	0.8	171.1	616.3	7.8	10
3rd Quad	2	603.8	824.1	0.9377	5.3	7.25	2	195	680.9	10	18
Max	2	792	872.8	1	9	11	2	322.5	1315.8	10	19

Sample statistics (minimum, first quartile, median, third quartile, and maximum values) for Kendall's coefficients and quadrant correlations that changed signs (inconsistent) or kept the same sign (consistent) between the randomly generated subset and the original data set. "Ties" is the number of ties (shared values) introduced by the method. "Median sample interval" is the median number of days between consecutive samples in the randomly selected data subset. "Sd sample interval" is the standard deviation (in days) of the number of days between samples in the data subset. "p value" is the p value for the null hypothesis that the Kendall's tau correlation is equal to 0. "n" is the number of samples in the randomly selected data subset. "n removed" is the number of samples removed from the original data to produce the data subsets for each well and parameter.

References

De Cicco, Laura A., Robert M. Hirsch, David Lorenz, William D. Watkins. 2018. dataRetrieval: R packages for discovering and retrieving water data available from Federal hydrologic web services. doi:10.5066/P9X4L3GE.

R Core Team. 2019. R: A language and environment for statistical computing. R Foundation for Statistical Computing, Vienna, Austria. https://www.R-project.org.

Smith, S. Jerrod, Stanley T. Paxton, Scott Christenson, Robert W. Puls, and James R. Greer. 2009. Flow Contribution and Water Quality with Depth in a Test Hole and Public-Supply Wells: Implications for Arsenic Remediation through Well Modification, Norman, Oklahoma, 2003-2006. U.S. Environmental Protection Agency. https://cfpub.epa.gov/si/si_public_record_report.cfm?Lab=NRMRL&dirEntryId=199062.

Tomlinson, Zachary. 2021. Impacts of well pumping on trace metals in the Central Oklahoma Aquifer, Norman, OK (Document No. 332554) [Master's Thesis, University of Oklahoma]. SHAREOK. https://shareok.org/handle/11244/332554.

U.S. Geological Survey. 2016. National Water Information System data available on the World Wide Web (USGS Water Data for the Nation), accessed 23 April, 2022. http://dx.doi.org/10.5066/F7P55KJN.

Wickham, Hadley, Romain François, Lionel Henry and Kirill Müller (2021). dplyr: A Grammar of Data Manipulation. R package version 1.0.4. https://CRAN.R-project.org/package=dplyr.

Wickham et al. 2019. Welcome to the tidyverse. *Journal of Open Source Software* 4 (43): 1686. https://doi.org/10.21105/joss.01686.
